# Feasibility of Remote Camera-Based Respiratory Motion Detection in Hospitalized Dogs Using the InsectEye Image-Analysis Application: A Pilot Study

**DOI:** 10.3390/vetsci13080781

**Published:** 2026-08-04

**Authors:** Akihiro Ohnishi, Mio Takeuchi, Ryo Sakaguchi, Runa Iwata, Taketoshi Asanuma, Yoshiki Itoh

**Affiliations:** 1 Department of Veterinary Science, Okayama University of Science, 1-3 Ikoinooka, Imabari 794-8555, Ehime, Japan; a-oonishi@ous.ac.jp (A.O.);; 2 Chushikoku Animal Eye Clinic, 3-9-3 Imazu-cho, Fukuyama 729-0111, Hiroshima, Japan

**Keywords:** dog, respiratory motion detection, noncontact monitoring, image analysis, infrared video, hospitalized animals, veterinary medicine, pilot study

## Abstract

Noncontact camera-based detection of respiratory motion may support low-burden surveillance of hospitalized dogs, particularly at night. This pilot study evaluated the feasibility of the InsectEye image-analysis application for detecting visually identifiable respiratory motion in selected infrared nighttime video segments. An initial parameter optimization was performed using 13 one-minute video segments from nine dogs. The selected analysis conditions were subsequently evaluated in Experiment 1 using five 5 min video recordings from four dogs. Experiment 2 further assessed respiratory rate estimation using the same validation dataset. InsectEye-detected events were compared with visually confirmed respiratory motion using time-synchronized review. Two criteria were assessed: Both-only, requiring detection of both inspiratory- and expiratory-related motion, and Both+Either, accepting detection of either or both phases. Detection performance improved when the threshold was reduced from 10 to 8, particularly with the 256 × 120 mesh. The 256-120_15_8 condition yielded the highest mean detection rate (87.93 ± 1.87%), fewer false-negative events, and shorter continuous non-detection duration, although false-positive detections increased. The Both+Either criterion performed better than the Both-only criterion. These findings support the feasibility of camera-based respiratory motion detection under selected favorable conditions, but larger studies using physiological respiratory reference methods are required before clinical monitoring or alerting applications can be established.

## Simple Summary

Monitoring breathing in hospitalized dogs can be difficult, particularly at night when continuous direct observation is not always possible. Camera-based image analysis may provide a noncontact method for detecting breathing-related body movements. In this pilot study, we evaluated whether the image-analysis application InsectEye could detect respiratory motion in selected nighttime video recordings of hospitalized dogs. Respiratory movements visually identified in the videos were compared with motion detected by the application using time-synchronized review. Detection performance was influenced by the analysis settings. A finer image-analysis mesh and a lower detection threshold improved the detection of respiratory motion and reduced missed respiratory events, although false-positive detections increased. Accepting detection of either inspiratory- or expiratory-related motion was more suitable than requiring detection of both phases. These findings suggest that camera-based image analysis may be feasible for detecting visually identifiable respiratory motion in selected video conditions. Further studies using larger numbers of dogs and physiological respiratory reference methods are needed before this approach can be used for clinical respiratory monitoring or alert systems.

## 1. Introduction

Respiratory monitoring is an important component of clinical observation in hospitalized dogs, particularly at night, when direct continuous observation is difficult. Respiratory status may change in association with postoperative recovery, discomfort, stress, history of sedation, or clinical deterioration. Although some patients clearly require full intensive care monitoring, others fall into an intermediate clinical category, in which the respiratory status remains a concern despite not meeting the threshold for ICU-level management. In such patients, clinically meaningful deterioration may occur without being immediately recognition, making practical surveillance of respiratory status an important aspect of inpatient care [1].

In veterinary hospitals, bridging this surveillance gap often depends on staff time, repeated direct observations, and manual reassessment of patients. However, this approach can be labor-intensive, particularly during overnight hospitalization. In addition, the frequent presence of staff near the patients may disturb the resting animals and potentially increase environmental stress. Thus, for patients who do not require full intensive care monitoring but still warrant closer surveillance, a practical dilemma may arise between closer observation and minimization of disturbances. Therefore, noncontact monitoring approaches that support respiratory surveillance without increasing handling or staff burden may be particularly valuable [1,2,3].

Conventional respiratory monitoring methods in veterinary medicine rely primarily on direct visual observation or contact-based devices. Direct observation is simple and intermittent and depends on staff availability. Contact-based monitoring can provide useful physiological information, but it may require sensor attachment, repeated handling, or equipment that is not always practical for routine use in non-ICU hospitalized patients [1,4]. Furthermore, the attachment of monitoring devices may itself disturb resting animals [2,4]. Therefore, noncontact approaches that allow surveillance without physically interfering with the patient may offer practical advantages in veterinary clinical settings [2,5].

In human medicine, camera- and image-analysis-based methods for noncontact physiological monitoring have attracted increasing interest [6,7]. However, noncontact respiratory monitoring in veterinary patients remains poorly established, and several challenges are specific to animal patients. Body position changes, variable thoracoabdominal visibility, coat characteristics, cage structure, and non-respiratory movements may all influence the detection of breathing-related motions from the video. Therefore, veterinary applications are not simple extensions of human-oriented approaches. Nevertheless, if respiratory motion can be detected under actual hospitalization conditions, video-based monitoring may be a useful adjunctive surveillance tool for veterinary inpatients [5,6,8].

The present pilot study evaluated the feasibility of remote camera-based respiratory motion detection in hospitalized dogs using the image-analysis application InsectEye. Archived infrared nighttime video recordings obtained from hospitalized dogs were analyzed to compare respiratory detection performance under different analysis conditions and to assess false-positive events, false-negative events, continuous non-detection duration, and agreement of respiratory rate estimation with visual assessment. We hypothesized that optimized analysis conditions would improve respiratory event detection and support the feasibility of this noncontact approach for respiratory surveillance in hospitalized dogs.

## 2. Materials and Methods

### 2.1. Study Design and Animals

This pilot study consisted of two sequential experiments using archived nighttime video recordings obtained from hospitalized client-owned dogs at the Veterinary Teaching Hospital of Okayama University of Science. The analyzed videos were originally recorded under the clinical study protocol used in a previous study of hospitalized dogs undergoing ophthalmic surgery. The present study was designed as a secondary analysis of those archived recordings. The included dogs were hospitalized for ophthalmic surgery either on the day before surgery or during the postoperative hospitalization period. In the original study population, dogs requiring nighttime care due to concurrent disease, dogs administered analgesics after 18:00, and dogs whose surgery was completed after 18:00 were excluded. In the present analysis, no additional analgesics were administered within 3 h before the analyzed recording period, and no drugs known to cause respiratory depression were used during the observation period to minimize pharmacological influences on respiratory motion and provide a consistent baseline for evaluation of the image-analysis algorithm. Written informed consent was obtained from the owner for data collection during the original study. The institutional ethics committee of the Okayama University of Science approved the ethical review for the secondary reuse of the archived recordings (approval no. 2021-0009).

An initial parameter optimization was performed using 13 one-minute video segments obtained from nine dogs to identify favorable analysis conditions. Based on these results, a validation dataset consisting of five 5 min video recordings from four dogs was selected. This validation dataset was subsequently used for Experiment 1, which evaluated respiratory detection performance under the selected analysis conditions, and for Experiment 2, which assessed respiratory rate estimation. Signalment data, including breed, sex, age, and body weight, were collected for all dogs.

### 2.2. Video Acquisition and Selection of Video Segments

The dogs were hospitalized in the intensive care unit (ICU) (ICU-MENIOS; Menix, Saitama, Japan). Depending on body size, the dogs were housed in cages measuring either 580 mm × 750 mm × 650 mm or 1160 mm × 750 mm × 650 mm, which allowed them to stand and move within the cage. Water was provided ad libitum during hospitalization.

Nighttime behavior during hospitalization was recorded using an infrared network camera (TS-WPTCAM2, I-O DATA, Kanazawa, Japan) positioned in front of the cage to capture the entire cage area. The videos were recorded at a resolution of 800 × 600 pixels and a frame rate of 30 frames/s.

For analysis, one-minute video segments were extracted from the archived nighttime recordings. Segments were selected from periods in which non-respiratory body movement was minimal and thoracic and/or abdominal respiratory motion was visually identifiable. Multiple segments from the same dog were included when different recording periods met the selection criteria. For the validation dataset, each selected recording consisted of a continuous 5 min nighttime video, from which one-minute segments and respiratory rate analysis windows were generated for the subsequent analyses.

### 2.3. Image Analysis Using InsectEye

**Figure 1:**
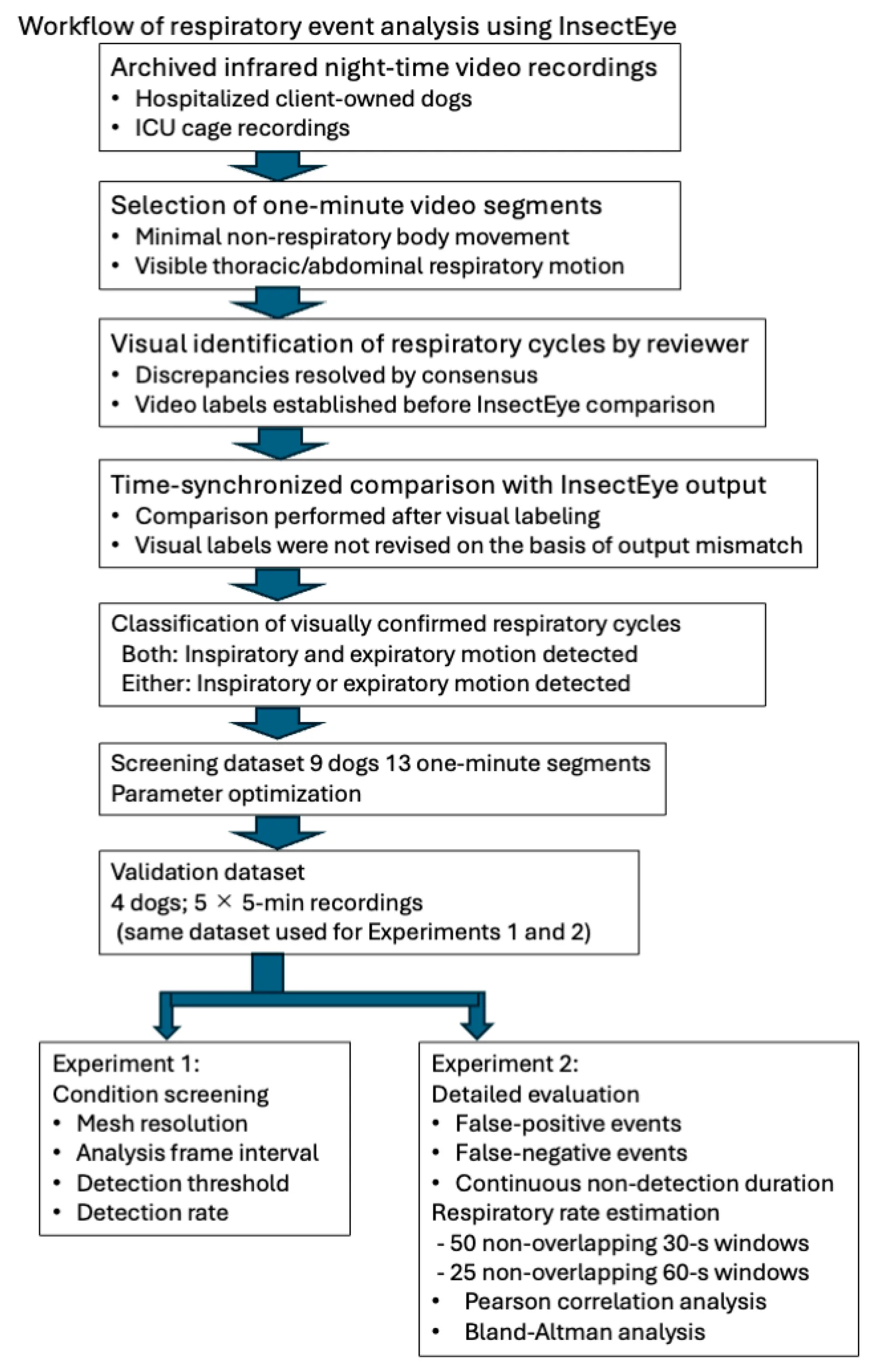
Workflow of respiratory event analysis using InsectEye. The diagram summarizes the study workflow, including the screening dataset used for parameter optimization, the validation dataset shared between Experiments 1 and 2, and the analyses performed in each experiment.

Selected video segments were analyzed using the image-analysis application InsectEye (Energy Gateway, Tokyo, Japan). The image-analysis workflow is illustrated in Figure 1.

In Experiment 1, respiratory detection performance was evaluated under different combinations of image-analysis parameters. Frame sampling interval refers to the interval at which video frames were sampled for analysis (i.e., every 10, 12, or 15 frames), rather than the frame rate itself. The evaluated parameters included mesh resolution (160 × 120 and 256 × 120), frame sampling intervals of every 10, 12, and 15 frames, and detection threshold. The initial comparison was performed using a detection threshold of 10. Based on the results, the detection threshold was subsequently lowered to 8 while maintaining the same mesh resolutions and frame sampling intervals to determine whether respiratory detection performance could be further improved.

In Experiment 2, the analysis conditions evaluated in Experiment 1 were further assessed with respect to false-positive events, and false-negative events, continuous non-detection duration, and respiratory rate estimation in order to characterize the practical performance of the application under the selected analysis settings.

### 2.4. Reference Assessment and Definition of Respiratory Events

The respiratory events detected by InsectEye were evaluated against visually confirmed respiratory motion in the corresponding video recordings. Visual assessment of the respiratory motion served as the reference standard for this pilot study.

**Figure 2:**
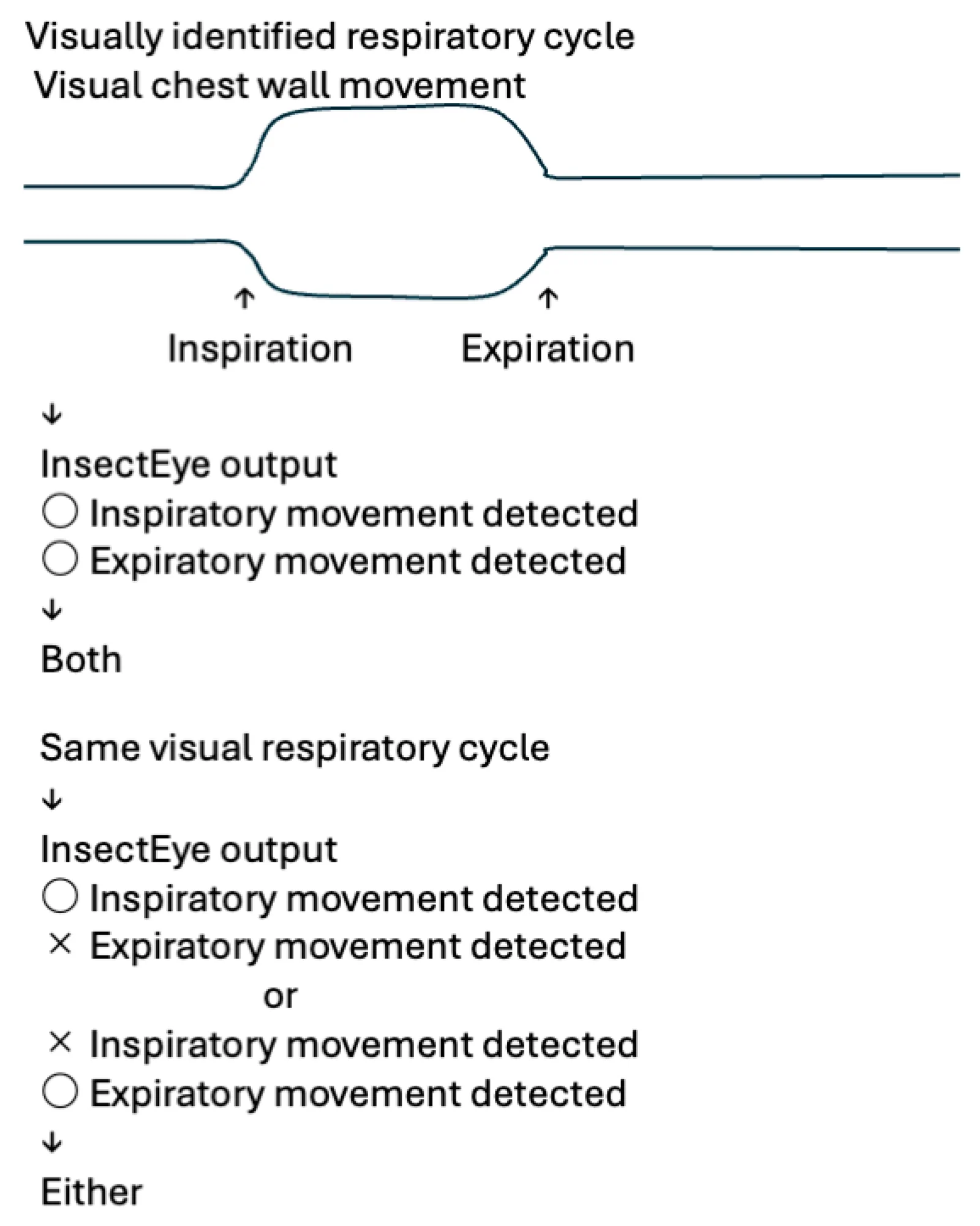
Schematic illustration of respiratory cycle classification. A visually confirmed respiratory cycle was classified as Both when InsectEye detected both inspiratory and expiratory chest wall movements within the same respiratory cycle. A respiratory cycle was classified as Either when only one respiratory phase (inspiration or expiration) was detected.

Respiratory motion is defined as the visible cyclic movement of the thoracic and/or abdominal body walls associated with breathing. Video recordings were reviewed manually by two investigators who identified the respiratory cycles based on visual observation alone. When discrepancies occurred between the two reviewers, the video recordings were rechecked and a consensus decision was made. The visually determined respiratory events were compared with the output data generated by InsectEye using a time-synchronized review. To minimize review bias, visually determined respiratory events were not reclassified based on the InsectEye output. The classification of a visually confirmed respiratory cycle according to the Both and Either criteria is illustrated schematically in Figure 2.

A visually confirmed respiratory cycle was classified as Both when InsectEye detected motions corresponding to both inspiratory and expiratory movements within the same respiratory cycle. A visually confirmed respiratory cycle was classified as Either when InsectEye detected motion corresponding to either the inspiratory phase or the expiratory phase alone.

For the performance evaluation, two detection criteria were applied. Under the Both-only criterion, only the respiratory cycles classified as Both were counted as detected events. Under the Both+Either criterion, respiratory cycles classified as either Both or Either were counted as detected events.

### 2.5. Outcome Measures

The outcome measures of this study were the detection rate, false-positive waveform count, false-negative waveform count, continuous non-detection duration, and descriptive agreement between the respiratory rates estimated by InsectEye and those determined by visual assessment.

In Experiment 1, the detection rates were compared across combinations of mesh resolution, frame sampling interval, and detection threshold to identify conditions with relatively favorable respiratory detection performance. In Experiment 2, the practical performance under the selected conditions was further evaluated in detail.

The detection rate was evaluated on a per-minute basis and was defined as the number of visually confirmed respiratory events detected by InsectEye divided by the total number of visually confirmed respiratory events within the same one-minute segment.

False-positive events were defined as waveform detections generated by InsectEye in the absence of visually confirmed respiratory motion and were expressed as the number of false-positive waveforms per minute. Because InsectEye is sensitive to motion, such detections may include non-respiratory body movements, such as brief jerkings or postural adjustments, in addition to algorithmic noise. False-negative events were defined as visually confirmed respiratory waveforms that were not detected by InsectEye and were expressed as the number of false-negative waveforms per minute.

The continuous non-detection duration was defined as the uninterrupted period during which no respiratory events were detected. This parameter was used as an index of temporal continuity for respiratory motion detection in this pilot study.

### 2.6. Respiratory Rate Assessment

The respiratory rate was calculated from the number of detected respiratory events and was expressed as breaths/min. Each 1 min video segment was divided into two non-overlapping 30 s windows or analyzed as a single 60 s window for respiratory rate estimation. Across all selected video segments in Experiment 2, this resulted in a total of 50 non-overlapping 30 s analysis windows and 25 non-overlapping 60 s analysis windows for each analysis condition. No sliding-window or overlapping segmentation approach was used. To evaluate the influence of the analysis settings on respiratory rate estimation, the respiratory rates obtained under each analysis condition were compared with the visually assessed respiratory rate.

### 2.7. Statistical Analysis

Statistical analyses were performed using GraphPad Prism (version 9.5.1; GraphPad Software, San Diego, CA, USA) and R (version 4.5.1; R Foundation for Statistical Computing, Vienna, Austria). Data are presented as mean ± standard deviation (SD), and statistical significance was set at *p* < 0.05.

For the analyses of respiratory detection rate, false-positive events, false-negative events, generalized linear mixed-effects models (GLMMs) were used to account for repeated measurements obtained from the same dogs and video segments. Dog identity and video window identity were included as random effects. Detection rate was analyzed using a binomial error distribution with a logit link function, whereas false-positive and false-negative event counts were analyzed using Poisson error distributions with a log link function. Type II Wald chi-square tests were used to evaluate the fixed effects of mesh resolution, frame sampling interval, and detection threshold. When significant main effects were detected, pairwise comparisons were performed using estimated marginal means with Tukey adjustment for multiple comparisons. Continuous non-detection duration was summarized descriptively because of the exploratory nature of this pilot study and the limited sample size.

Correlations between the respiratory rates obtained using InsectEye and those determined by visual assessment were evaluated using Pearson’s correlation analysis. Agreement between the two methods was further assessed using Bland–Altman analysis, in which the mean difference (bias) and 95% limits of agreement were calculated.

### 2.8. Use of Generative Artificial Intelligence

During the preparation of this manuscript, the authors used ChatGPT (OpenAI, San Francisco, CA, USA) solely to improve the clarity and readability of the English text and to refine manuscript organization. Generative AI was not used for study design, data collection, data generation, statistical analysis, figure generation, or interpretation of the results. All scientific content and final editorial decisions were reviewed and verified by the authors.

## 3. Results

### 3.1. Experiment 1: Comparison of Detection Rates Across Analysis Conditions

**Figure 3:**
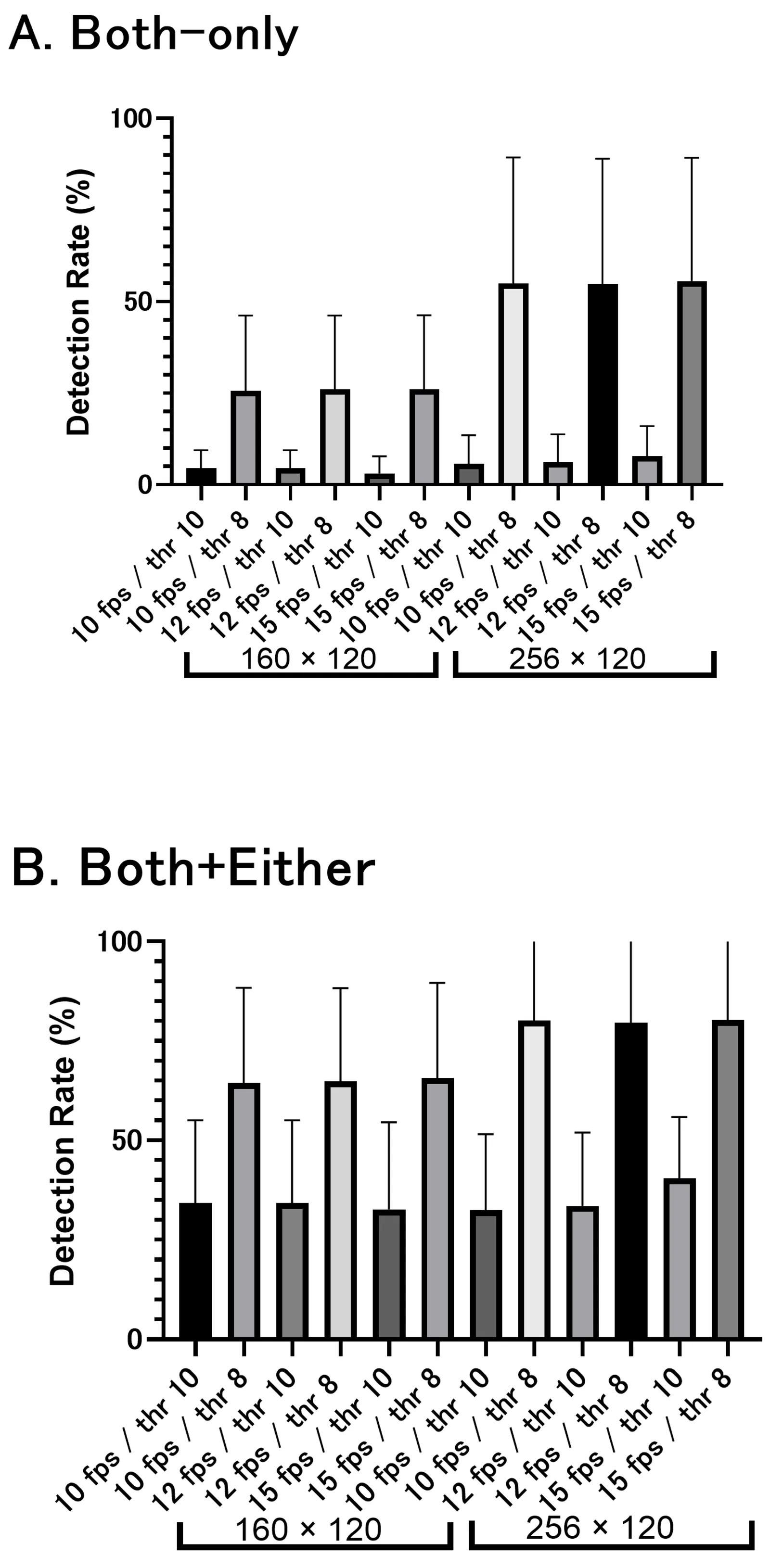
Detection rates across analysis conditions under the Both-only and Both+Either criteria. (**A**) Detection rates under the Both-only criterion. (**B**) Detection rates under the Both+Either criterion. Detection rates were compared across combinations of mesh resolution, frame sampling interval, and detection threshold using generalized linear mixed models with dog ID included as a random effect, followed by Tukey-adjusted post hoc comparisons. Bars represent mean ± SD. Detection rates were compared using generalized linear mixed models with dog ID included as a random effect, followed by Tukey-adjusted post hoc comparisons. Exact adjusted *p* values for all pairwise comparisons are provided in Supplementary Table S1.

Respiratory detection rates under the Both-only criterion were analyzed using a generalized linear mixed-effects model that accounted for repeated measurements from the same dogs and video segments. Mesh resolution (χ^2^ = 219.98, *p* < 0.001) and detection threshold (χ^2^ = 591.44, *p* < 0.001) significantly affected respiratory detection rate, whereas frame sampling interval had no significant effect (χ^2^ = 0.15, *p* = 0.711). Post hoc comparisons showed significantly higher detection rates with the 256 × 120 mesh than with the 160 × 120 mesh (*p* < 0.001) and with a detection threshold of 8 than with a threshold of 10 (*p* < 0.001). No significant differences were observed among the three frame intervals (every 10, 12, and 15 frames) (Figure 3A).

Similar results were obtained using the Both+Either criterion. Mesh resolution (χ^2^ = 42.69, *p* < 0.001) and detection threshold (χ^2^ = 586.65, *p* < 0.001) significantly affected respiratory detection rate, whereas frame sampling interval had no significant effect (χ^2^ = 1.5, *p* = 0.9295). Post hoc comparisons likewise demonstrated significantly higher detection rates with the 256 × 120 mesh than with the 160 × 120 mesh (*p* < 0.001) and with a detection threshold of 8 than with a threshold of 10 (*p* < 0.001). No significant differences were observed among the three frame intervals (Figure 3B).

### 3.2. Experiment 2: False-Positive and False-Negative Events

**Figure 4:**
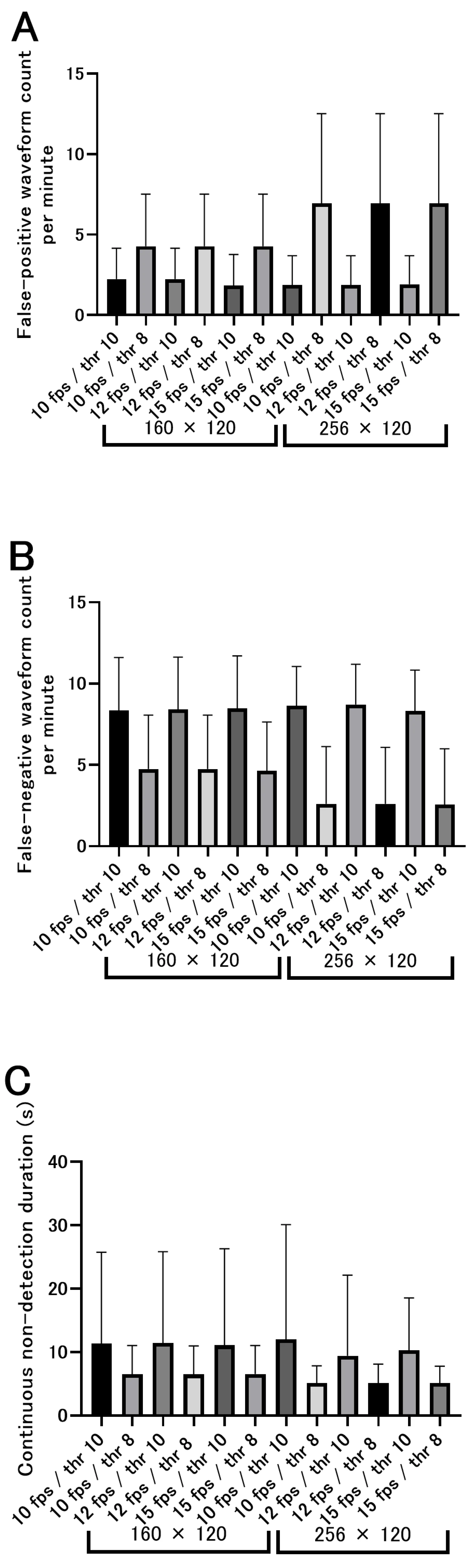
False-positive waveform counts, false-negative waveform counts, and continuous non-detection duration across analysis conditions. (**A**) False-positive waveform count per minute. (**B**) False-negative waveform count per minute. (**C**) Continuous non-detection duration. Bars represent mean ± SD.

The number of false-positive respiratory events was analyzed using a generalized linear mixed-effects model. Mesh resolution (χ^2^ = 29.91, *p* < 0.001) and detection threshold (χ^2^ = 238.06, *p* < 0.001) significantly affected the number of false-positive events, whereas frame sampling interval had no significant effect (χ^2^ = 0.14, *p* = 0.931). Post hoc comparisons showed significantly fewer false-positive events with the 160 × 120 mesh than with the 256 × 120 mesh (*p* < 0.001) and with a detection threshold of 10 than with a threshold of 8 (*p* < 0.001). No significant differences were observed among the three frame intervals (Figure 4A).

The number of false-negative respiratory events was also analyzed using a generalized linear mixed-effects model. Mesh resolution (χ^2^ = 12.23, *p* < 0.001) and detection threshold (χ^2^ = 273.55, *p* < 0.001) significantly affected the number of false-negative events, whereas frame sampling interval had no significant effect (χ^2^ = 0.13, *p* = 0.937). Post hoc comparisons showed significantly fewer false-negative events with the 256 × 120 mesh than with the 160 × 120 mesh (*p* < 0.001) and with a detection threshold of 8 than with a threshold of 10 (*p* < 0.001). No significant differences were observed among the three frame intervals (Figure 4B).

### 3.3. Experiment 2: Continuous Non-Detection Duration

Continuous non-detection duration decreased after lowering the detection threshold under both mesh settings (Figure 4C). In the 160 × 120 conditions, the mean continuous non-detection duration decreased from 11.09–11.46 s at threshold 10 to 6.48–6.54 s at threshold 8. In the 256 × 120 conditions, the corresponding values decreased from 9.41–12.01 s to 5.10–5.11 s. Among the evaluated conditions, the 256 × 120_15_8 setting showed a mean continuous non-detection duration of 5.11 ± 2.67 s, representing the shortest duration observed.

### 3.4. Experiment 2: Correlation Between InsectEye-Derived and Visually Assessed Respiratory Rates

**Figure 5:**
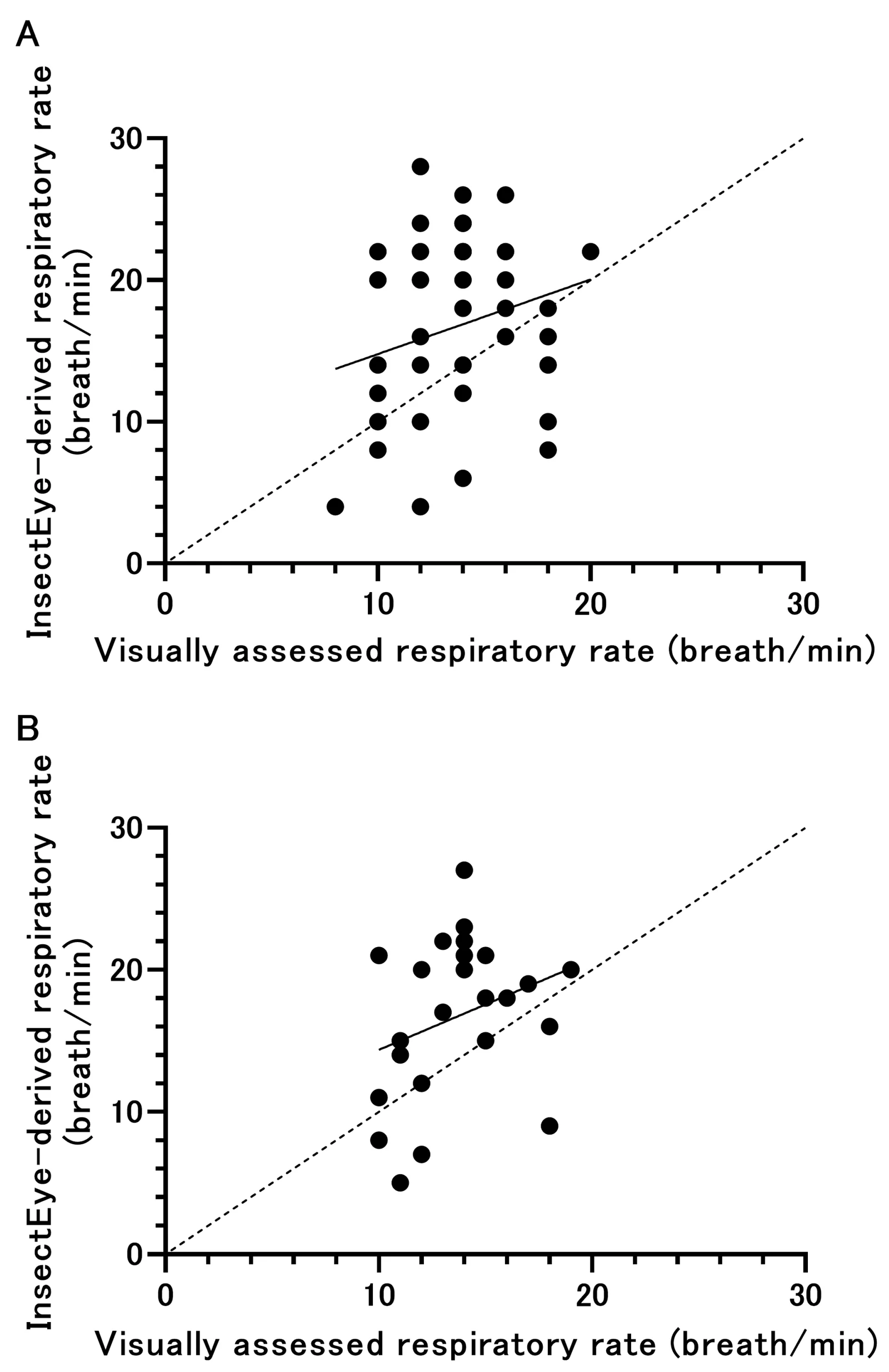
Correlation between InsectEye-derived and visually assessed respiratory rates under the representative optimized analysis condition (256-120_15_8) using the Both+Either criterion; (**A**) 30 s analysis window (*n* = 50 non-overlapping analysis windows); (**B**) 60 s analysis window (n = 25 non-overlapping analysis windows). Each point represents one non-overlapping analysis window generated from five selected 5 min video recordings obtained from four dogs. Multiple analysis windows were generated from individual video recordings; therefore, repeated observations from the same dog are included. The dashed line represents the identity line (y = x), and the solid line represents the linear regression line.

Under the Both+Either criterion, InsectEye-derived respiratory rates showed significant positive correlations with visually assessed respiratory rates, whereas correlations under the Both-only criterion were generally weaker (Figure 5A,B). Respiratory rate analyses were performed using all non-overlapping analysis windows generated from the five selected 5 min video recordings, resulting in 50 analysis windows for the 30 s analysis and 25 analysis windows for the 60 s analysis under each analysis condition (Figure 1). In the 30 s analysis window, the strongest correlation under the Both+Either criterion was observed for the 256 × 120_15_8 condition (r = 0.6652, 95% CI 0.4699–0.7984, *p* < 0.0001). Similar significant correlations were observed for the 256 × 120_10_8 (r = 0.6240, *p* < 0.0001) and 256 × 120_12_8 (r = 0.6369, *p* < 0.0001) conditions.

In the 60 s analysis window, significant positive correlations were observed across all tested conditions under the Both+Either criterion (r = 0.4283–0.7198). The highest correlation was again obtained for the 256 × 120_15_8 condition (r = 0.7198, 95% CI 0.4459–0.8705, *p* < 0.0001), followed by the 256 × 120_12_8 (r = 0.6985, *p* = 0.0001) and 256 × 120_10_8 (r = 0.6743, *p* = 0.0003) conditions.

In contrast, under the Both-only criterion, significant correlations were limited to the 256 × 120 conditions with a threshold of 8 in the 30 s analysis window (r ≈ 0.37, *p* < 0.05), whereas no significant correlations were observed in the 60 s analysis window.

### 3.5. Experiment 2: Agreement Analysis for the Optimized Condition

Because the 256 × 120_15_8 condition showed the highest respiratory detection rate and strong performance across the evaluated metrics, agreement between InsectEye-derived and visually assessed respiratory rates was further evaluated using Bland–Altman analysis.

**Figure 6:**
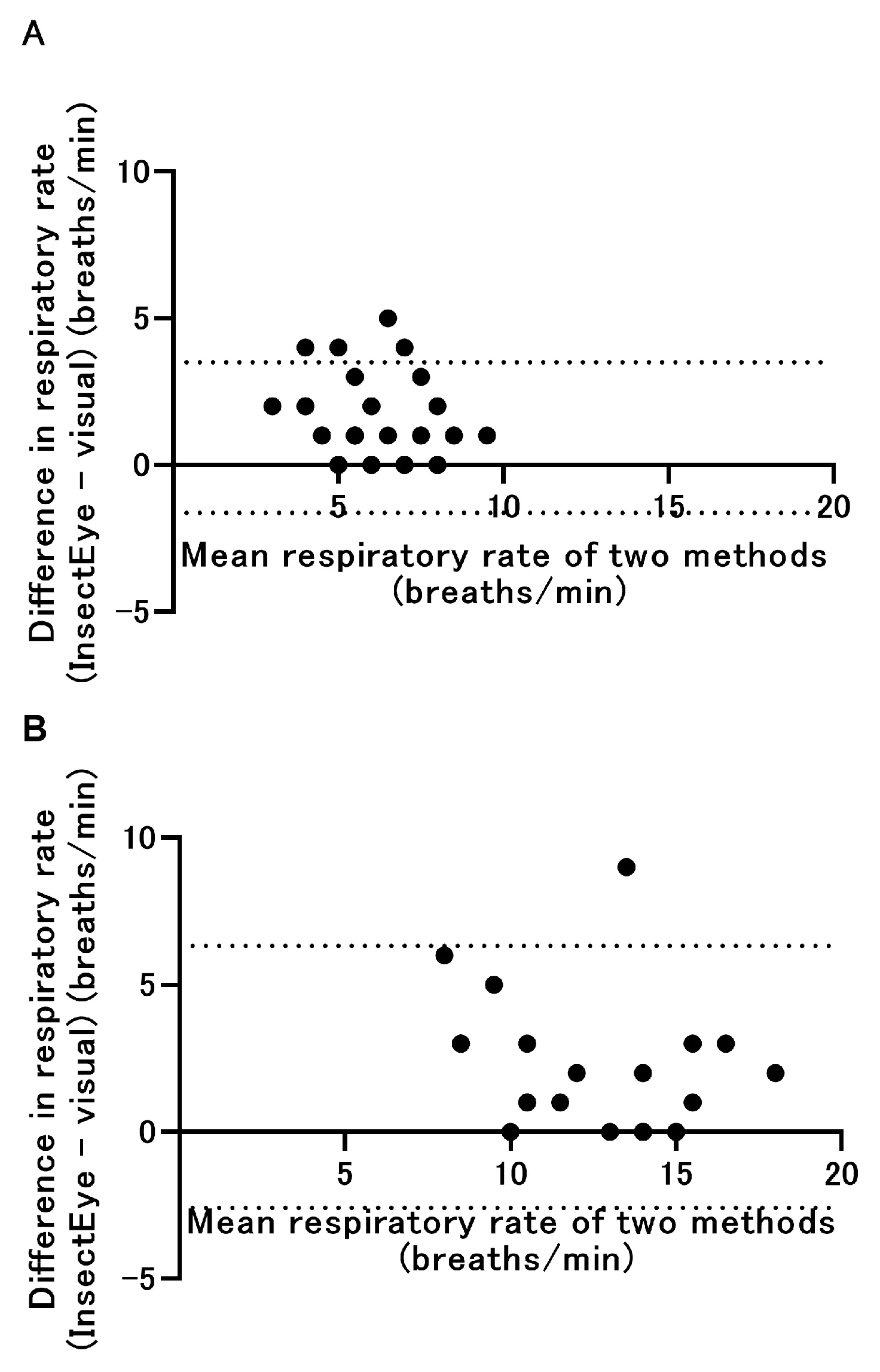
Bland–Altman analysis of agreement between InsectEye-derived and visually assessed respiratory rates under the representative optimized analysis condition (256-120_15_8) using the Both+Either criterion; (**A**) 30 s analysis window (n = 50 non-overlapping analysis windows); (**B**) 60 s analysis window (n = 25 non-overlapping analysis windows). Each point represents one non-overlapping analysis window generated from five selected 5 min video recordings obtained from four dogs. Consequently, multiple observations were obtained from the same dogs and video recordings. The solid line indicates the mean difference (bias), and the dotted lines indicate the 95% limits of agreement.

In the 30 s analysis window, the mean bias was 0.94 breaths/min, with 95% limits of agreement ranging from −1.63 to 3.51 breaths/min. In the 60 s analysis window, the mean bias was 1.88 breaths/min, with 95% limits of agreement ranging from −2.58 to 6.33 breaths/min (Figure 6A,B).

Under the 256 × 120_15_8 condition, the respiratory detection rate was 87.93 ± 1.87%, the mean number of false-negative events was 2.08 events/min, and the mean continuous non-detection duration was 5.11 ± 2.67 s.

## 4. Discussion

This pilot study evaluated the feasibility of camera-based detection of respiratory motion in selected nighttime video segments obtained from hospitalized dogs using the InsectEye image-analysis application. The present study demonstrated that respiratory motion identified by visual review could also be detected by InsectEye under selected, relatively favorable video conditions. Among the tested settings, the 256 × 120 mesh combined with a threshold of 8 provided the most favorable overall balance between respiratory detection rate, false-negative events, and continuous non-detection duration, although this setting also increased false-positive detections.

From a clinical perspective, the noncontact nature of this approach is particularly attractive [9]. The respiratory status is often of interest to hospitalized dogs, even in cases that do not require full intensive care monitoring. In such patients, a camera-based method may allow observation of the respiratory status without attaching sensors to the patient, disturbing the animal during rest, or requiring additional handling [1,2,7]. This may be especially advantageous during nighttime hospitalization, when practical, low-burden monitoring approaches are desirable. Therefore, the present findings suggest potential utility of this approach as an adjunctive surveillance tool in routine clinical settings.

An important finding of this study was that the Both+Either criterion was more suitable for respiratory event capture and respiratory rate estimation than the stricter Both-only criterion. Under the Both-only criterion, a respiratory cycle was counted as detected only when InsectEye recognized both inspiratory- and expiratory-phase-related motion within the same visually confirmed cycle. By contrast, the Both+Either criterion also accepted cycles in which only one respiratory phase was detected. Respiratory motion in hospitalized dogs may be subtle, partially obscured, or affected by body position and minor movement, requiring complete detection in both phases, which appears to be overly restrictive in this pilot setting. In practical surveillance applications, detection of either inspiratory- or expiratory-related motion may be sufficient to indicate ongoing breathing activity. This likely explains why the Both+Either criterion achieved higher respiratory detection rates, fewer false-negative events, shorter continuous non-detection durations, and stronger correlations with visually assessed respiratory rates than the stricter Both-only criterion.

Lowering the detection threshold from 10 to 8 substantially improved the detection performance, particularly under the 256 × 120 mesh conditions. This improvement was accompanied by a marked reduction in false-negative events, indicating that fewer visually confirmed respiratory cycles were missed at the lower threshold setting. Simultaneously, false-positive detections increased, especially under the 256 × 120 threshold 8 conditions. These findings indicate a clear trade-off between sensitivity and specificity-like behavior in this system. Lowering the threshold reduced missed respiratory events and shortened non-detection periods but also made the system more responsive to non-respiratory motion. Therefore, some false-positive detections may have reflected not only algorithmic noise but also non-respiratory body movements, such as brief jerking, postural adjustment, or other subtle thoracoabdominal motions that were not classified as a visually confirmed respiratory event. In the hospitalized setting, such minor movements are difficult to eliminate completely. In future clinical surveillance systems, excessive false-positive detections could increase the frequency of unnecessary alerts and require additional manual verification by clinical staff. Therefore, further optimization of the detection algorithm will be necessary to balance respiratory detection performance with an acceptable false-positive rate before automated alert functions can be implemented in routine clinical practice. Accordingly, the optimized condition should be interpreted as representing the most favorable overall balance rather than a perfect discrimination between respiratory and non-respiratory motions.

An additional important observation was that within the 256 × 120 threshold 8 group, no significant differences were detected among the 10-, 12-, and 15-frame sampling intervals for the detection rate, false-positive count, or false-negative count. This suggests that once the mesh resolution and threshold were optimized, further increasing the frame interval settings within the tested range did not substantially alter the overall monitoring performance. From a practical perspective, this finding may be useful when selecting analysis conditions that balance the computational efficiency and respiratory detection performance.

The continuous non-detection duration was another important finding of this study. In addition to respiratory rate estimation, remote monitoring systems should ideally maintain the temporal continuity of respiratory event capture. Under optimized conditions, the prolonged intervals without detected respiratory motion became shorter, reaching approximately 5 s in the 256 × 120 threshold 8 conditions. From a practical monitoring perspective, this suggests that the optimized setting may be better suited for surveillance-oriented applications than settings with longer uninterrupted non-detection periods. In other words, the present system may have value not only for respiratory rate estimation but also for continuous observation of whether respiratory motion is being detected over time.

This observation is related to the potential future use of this system for apnea-related alerts. In the present study, the Both+Either criterion captured at least one respiratory-phase-related motion in a high proportion of visually confirmed respiratory cycles, and the optimized conditions reduced the prolonged non-detection intervals. These characteristics may be favorable for identifying interruptions in detectable breathing-related motion. However, the present study did not directly evaluate true apnea events or breath-holding episodes. Therefore, although the findings suggest the potential applicability to apnea-related alerting, dedicated validation under apnea-relevant conditions is required before any clinical alerting function can be established [3,4].

Agreement analysis for the optimized condition showed a relatively small mean bias and narrower limits of agreement in the 30 s window, whereas the 60 s window demonstrated a larger systematic bias and wider limits of agreement. Simultaneously, the correlation coefficients tended to be higher in the 60 s window than in the 30 s window. These findings indicate that correlation and agreement describe different aspects of performance. Although the 60 s window showed a stronger linear association with visually assessed respiratory rates, the 30 s window demonstrated better agreement with smaller bias and narrower limits of agreement. Therefore, the choice of analysis window should depend on the intended clinical application and whether greater emphasis is placed on agreement or linear association with the reference measurements.

Several limitations should be emphasized. First, the number of dogs and video segments was small, particularly in Experiment 2. Second, although repeated measurements from the same dogs and video windows were appropriately accounted for using generalized linear mixed-effects models, the number of dogs and analyzed video segments remained limited. Therefore, these findings should be interpreted as exploratory and require validation in a larger population. Third, visual assessment of video recordings was used as the reference method rather than a physiological respiratory monitoring technique such as capnography or respiratory inductance plethysmography. Visual assessment primarily reflects thoracoabdominal motion rather than physiological airflow or tidal volume and therefore cannot be regarded as a physiological gold standard for respiration [10]. Furthermore, interpretation of infrared video recordings is inherently subject to observer-related uncertainty. Subtle non-respiratory body movements, such as minor postural adjustments or other small body-wall movements, may occasionally be difficult to distinguish from true respiratory motion, potentially introducing uncertainty into the visual reference standard. Consequently, the present study evaluated agreement with expert visual assessment rather than the physiological accuracy of respiratory events. Future studies incorporating physiological reference methods will be necessary to further validate the clinical performance of the proposed system. Fourth, video segments were intentionally selected from periods with minimal overall body movement and visually identifiable respiratory motion. As a result, the findings likely represent performance under relatively favorable conditions and should not be generalized to all nighttime hospitalization scenarios. Fifth, false-positive detections in this study should not be interpreted solely as algorithmic errors, because some may have reflected subtle non-respiratory or ambiguous body-wall movements. Finally, dogs that had recently received analgesics or drugs known to cause respiratory depression were excluded to minimize pharmacological influences on respiratory motion and to establish a consistent baseline for algorithm evaluation. Consequently, the performance of the proposed algorithm has not yet been validated in sedated, analgesic-treated, or critically ill patients, whose respiratory patterns may differ substantially from those evaluated in this study. Further studies are warranted to assess its performance and clinical applicability in these patient populations.

Despite these limitations, this study provides initial evidence that noncontact respiratory monitoring using archived infrared videos and image analysis is technically feasible in hospitalized dogs. The study design also had a practical advantage in that visually determined respiratory events were established before comparison with the InsectEye output and were not retrospectively revised based on output mismatch, thereby reducing the risk of review bias in the reference labeling process. In addition, the present findings were obtained under real nighttime hospitalization conditions rather than in an artificial laboratory setting, which supports the potential clinical relevance of this approach.

Future studies should include a larger number of dogs and video segments and should compare InsectEye-derived respiratory detection with physiological respiratory reference methods [9]. Additional evaluation across different body positions, coat types, degrees of body movement, and clinical conditions would also be valuable. From a translational perspective, further development of this approach may support practical remote respiratory surveillance in hospitalized veterinary patients using the existing infrared camera systems already installed in veterinary hospitals, potentially allowing clinicians to monitor selected non-ICU patients without adding physical sensors or disturbing resting animals [4].

## 5. Conclusions

In conclusion, this pilot study suggests that InsectEye may be feasible for noncontact detection of visually identifiable respiratory motion in selected nighttime video segments from hospitalized dogs. Among the tested analysis settings, the 256 × 120 mesh with a threshold of 8 showed the most favorable overall performance, with higher detection rates, fewer false-negative events, and shorter continuous non-detection durations, although false-positive detections increased. The Both+Either criterion provided more favorable respiratory detection performance than the stricter Both-only criterion for surveillance-oriented monitoring in this pilot setting. Larger studies using physiological respiratory reference methods are required before this approach can be considered a validated clinical respiratory monitoring tool.

## Data Availability

The original contributions presented in this study are included in the article/Supplementary Material. Further inquiries can be directed to the corresponding author.

## Supplementary Materials

The following supporting information can be downloaded at https://www.mdpi.com/article/10.3390/vetsci13080781/s1, Table S1. Statistical results for respiratory event detection. (A) Overall effects of explanatory variables on respiratory event detection under the Both-only and Both+Either criteria, as determined by generalized linear mixed models. (B) Tukey-adjusted pairwise comparisons of detection rates for each explanatory variable under the Both-only and Both+Either criteria. Dataset S1. Raw dataset used for the generalized linear mixed model analyses. The dataset includes respiratory event detection results, analysis conditions, Dog ID, Window ID, and visually assessed reference data used for the statistical analyses presented in this study.
