# Supplementary material for: Feasibility of Remote Camera-Based Respiratory Motion Detection in Hospitalized Dogs Using the InsectEye Image-Analysis Application: A Pilot Study
Source: Vet Sci. 2026 Aug 4;13(8):781. doi: 10.3390/vetsci13080781 (PMC13517811; doi:10.3390/vetsci13080781)
Supplement: Supplementary file 1 [file vetsci-13-00781-s001.zip › Table S1.pdf]

Table S1. Overall effects of explanatory variables and Tukey-adjusted pairwise comparisons for detection rates estimated using generalized linear mixed models. The models included dog ID and analysis window ID as random effects. Pairwise P values were adjusted using Tukey's method for multiple comparisons.

A. Overall effects of explanatory variables on detection rate estimated by the generalized linear mixed model.

Criterion	Factor	χ^2	df	*P* value
Both-only	Mesh resolution	219.98	1	<0.0001
	Frame interval	0.14	2	0.7111
	Detection threshold	591.45	1	<0.0001
Both+Either	Mesh resolution	42.69	1	<0.0001
	Frame sampling interval	0.15	2	0.9295
	Detection threshold	586.65	1	<0.0001

B. Tukey-adjusted pairwise comparisons for detection rate.

Criterion	Factor	Comparison	Adjust *P* value
Both-only	Mesh resolution	160-120 vs 256-120	0.0005
	Frame interval	10 vs 12	0.9946
		10 vs 15	0.5818
		12 vs 15	0.6449
	Detection threshold	8 vs 10	<0.0001
Both+Either	Mesh resolution	160-120 vs 256-120	<0.0001
	Frame sampling interval	10 vs 12	0.9953
		10 vs 15	0.5077
		12 vs 15	0.5655
	Detection threshold	8 vs 10	<0.0001
